# Coupling and heterogeneity modulate pacemaking capability in healthy and diseased two-dimensional sinoatrial node tissue models

**DOI:** 10.1371/journal.pcbi.1010098

**Published:** 2022-11-21

**Authors:** Chiara Campana, Eugenio Ricci, Chiara Bartolucci, Stefano Severi, Eric A. Sobie

**Affiliations:** 1 Department of Pharmacological Sciences, Icahn School of Medicine at Mount Sinai, New York, New York, United States of America; 2 Department of Electrical, Electronic, and Information Engineering "Guglielmo Marconi", University of Bologna, Cesena, Italy; University of Virginia, UNITED STATES

## Abstract

Both experimental and modeling studies have attempted to determine mechanisms by which a small anatomical region, such as the sinoatrial node (SAN), can robustly drive electrical activity in the human heart. However, despite many advances from prior research, important questions remain unanswered. This study aimed to investigate, through mathematical modeling, the roles of intercellular coupling and cellular heterogeneity in synchronization and pacemaking within the healthy and diseased SAN. In a multicellular computational model of a monolayer of either human or rabbit SAN cells, simulations revealed that heterogenous cells synchronize their discharge frequency into a unique beating rhythm across a wide range of heterogeneity and intercellular coupling values. However, an unanticipated behavior appeared under pathological conditions where perturbation of ionic currents led to reduced excitability. Under these conditions, an intermediate range of intercellular coupling (900–4000 MΩ) was beneficial to SAN automaticity, enabling a very small portion of tissue (3.4%) to drive propagation, with propagation failure occurring at both lower and higher resistances. This protective effect of intercellular coupling and heterogeneity, seen in both human and rabbit tissues, highlights the remarkable resilience of the SAN. Overall, the model presented in this work allowed insight into how spontaneous beating of the SAN tissue may be preserved in the face of perturbations that can cause individual cells to lose automaticity. The simulations suggest that certain degrees of gap junctional coupling protect the SAN from ionic perturbations that can be caused by drugs or mutations.

## Author summary

In the mammalian heart, a small region of cells known as the sinoatrial node drives electrical activity in the remainder of the organ, and this mechanism avoids failure through the lifetimes of most individuals. It is unclear how such robust function can be maintained in the face of perturbations such as drugs, disease, and alterations in physiological state. Here we explored, through mathematical modeling of sinoatrial nodal tissue, how heterogeneity between cells and alterations in intercellular coupling affect the collective electrical behavior of the tissue. Our simulations showed that normal, synchronous electrical activity could be maintained under a wide range of conditions, even when a substantial percentage of the cells lost their capacity for autonomous electrical excitation. Under simultated disease conditions, we found that only reduced coupling between cells allowed for collective electrical activity, contrary to expectations that stronger coupling would always be beneficial. Together, these computational results provide insight into how this vitally important region of the heart maintains robust activity under a wide range of conditions.

## Introduction

Understanding the mechanisms that coordinate the spontaneous firing of the sinoatrial node (SAN) has long been an issue of great interest in cardiac electrophysiology. After early studies believed that a single pacemaker region drives the entire SAN, more recent research has shown that the heartbeat originates from the coordination of a complex structure [1]. Many studies have worked to unravel the basis of this coordination, through both experiments [2–4] and mathematical modeling [5–7]. Despite the many insights obtained by these studies, important questions remain unresolved, particularly with respect to how heterogeneity between SAN myocytes and inter-cellular coupling combine to influence coordinated beating in tissue. For example, although it has recently been shown experimentally that not all SAN cells fire spontaneously when they are enzymatically isolated [8–10], we do not know how non-firing cells behave when they are electrically coupled in tissue, nor how the percentage of non-firing cells influences the overall electrical activity of the SAN.

Multiple mathematical models exist in the literature that describe the electrophysiology of isolated SA nodal myocytes [11,12]. Most of these have been developed on the basis of data obtained in animal models, especially rabbits [13,14], but a model based on human data has been published more recently [15]. Although it is obviously helpful to have multiple tools available for computational analyses, a question that commonly arises in such circumstances is the extent to which the behavior observed in a particular model is generalizable. On the other hand, when similar trends are seen across multiple mathematical representations, this can provide confidence in the model predictions [16–18].

In this investigation, we performed cellular and tissue simulations to examine how heterogeneity between SAN myocytes and intercellular coupling influence the coordination of beating within the SA node. The main goals were to: i) assess the effect of cellular heterogeneity in isolated SAN cells; ii) gain mechanistic insight into how electrical coupling between SAN cells modulates pacemaker activity at different levels of heterogeneity; and iii) investigate how simulated Sinus Node Disease (SND) influences SAN automaticity. Heterogeneous populations of SAN myocytes were generated at several levels of variability, and physiological behavior was simulated in both isolated cells and 2-dimensional tissue. Major results of the simulations were: i) cellular heterogeneity increases AP frequency and duration as well as the percentage of “dormant” cells, with remarkable consistency between three SAN myocyte models [13–15]; ii) intercellular coupling allows the cells to synchronize the beating rate in all conditions, except when heterogeneity is large and coupling between myocytes is weak; and iii) blockade of particular ionic currents leads to a loss of robustness in which coordinated beating of the tissue fails at high and low coupling but can be maintained within a narrow range of intermediate coupling values. Overall, these simulations provide insight into the conditions that promote synchronized beating in the SAN, and how this can be maintained in the presence of heterogeneity.

## Methods

### Study design

**Fig 1:**
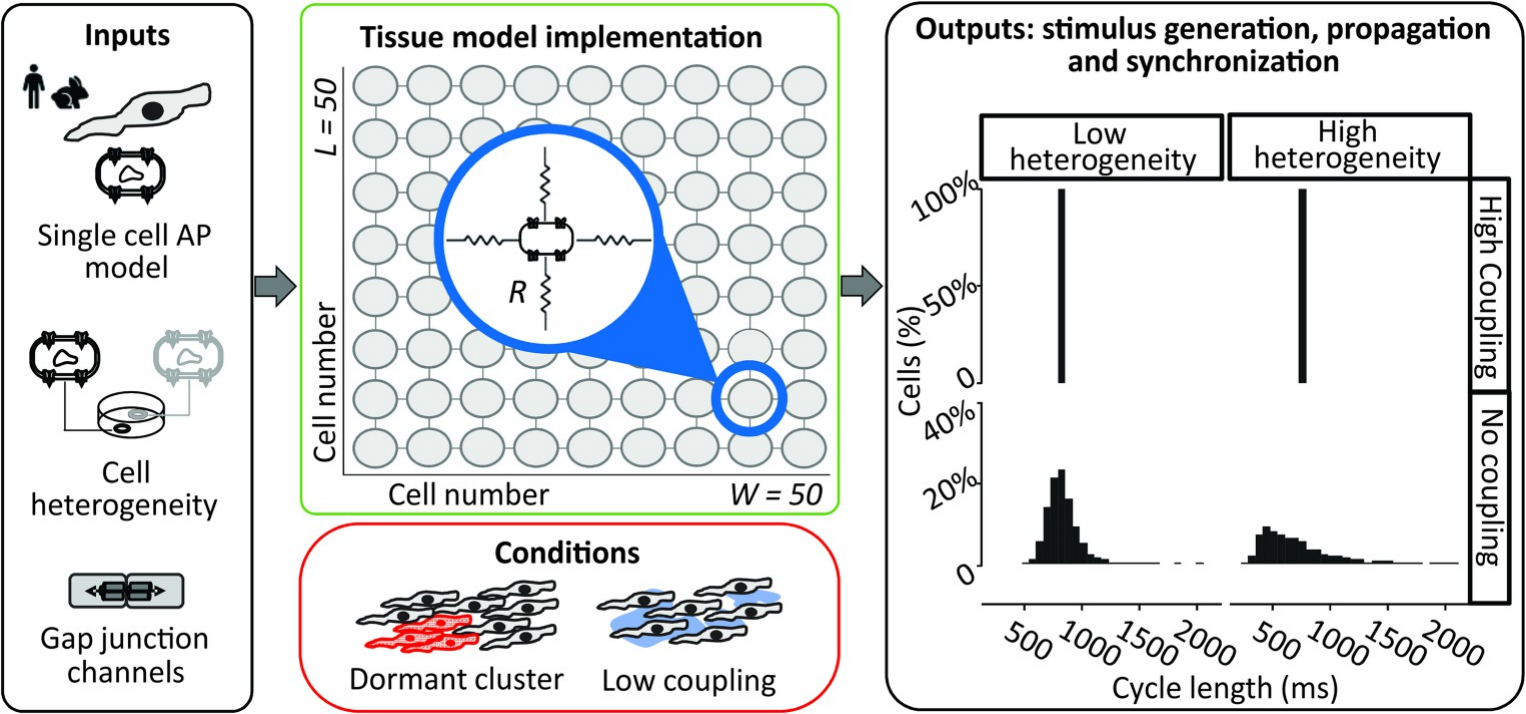
Schematic of multicellular study design. A multiscale mathematical modeling approach was employed to study the mechanisms of sinoatrial node excitability. The effect of cell-to-cell coupling and cellular heterogeneity on tissue synchronization were evaluated in both healthy sinoatrial nodes and those that mimicked Sinus Node Disease. Blue inset in central top panel shows that cells were connected to 4 neighbors using ohmic resistances that modeled gap junctions between adjacent myocytes.

The goal of our study was to analyze, through mechanistic simulations, how heterogeneity between cells and gap junctional coupling influence the automaticity of the sinoatrial node and entrainment of the Action Potential (AP). As schematically shown in Fig 1, a two-dimensional tissue model was developed using, as the building block, models of the isolated SAN cell of different species (human and rabbit). For human SAN, the recent Fabbri et al. [15] model was used, whereas for rabbit SAN, both the Maltsev-Lakatta [13] and Severi et al. [14] models were considered. As shown in the expanded section of the center panel, each myocyte is electrically connected to its neighbors through gap junctional resistances. These connections may result from connexin 43 or connexin 45 isoforms, or both, with the composition of SAN gap junctions still a topic of active debate [19,20]. This tissue model can then be used to simulate normal beating in the well-coupled SAN and to determine the effect of structural remodeling due to conditions such as reduced coupling (mimicking diffuse fibrosis [21,22]) and clusters of non-spontaneous (“dormant”) cells [8–10].

### Modeling heterogeneous populations of SAN cells

Heterogeneity between myocytes was simulated in each model by varying the maximal conductances of the ionic currents such that each current’s baseline conductance was multiplied by a random scale factor chosen from a lognormal distribution [23,24]. Five different values of the lognormal distribution shape factor (σ; from 0.1 to 0.5) were used to account for different levels of heterogeneity. Measurements from relatively large numbers of SAN myocytes (30–130) have revealed considerable heterogeneity in ionic current magnitudes, up to a 10-fold difference between cells with the largest and those with the smallest ionic currents [25]. As a lognormally-distributed random variable with σ = 0.5 shows a ratio of approximately 5 between the 95^th^ and the 5^th^ percentiles, these simulations may in fact underestimate true biological variability.

The purpose of creating heterogeneous populations of cells was three-fold. First, we used these populations to run a sensitivity analysis where the contributions of individual ionic currents on the cell’s automaticity were evaluated with a logistic regression model [26,27]. Second, isolated cell simulations were performed to assess the effects of σ on the AP parameters and on each model’s robustness (that is, how many cells showed spontaneous beating after parameter randomization). Third, the cellular populations were used to create the two-dimensional propagation model in an attempt to recapitulate a small part of the complexity characteristic of the SAN structure. In particular, we aim to compare the behavior of isolated and coupled cells to gain a mechanistic understanding of how coupling modulates the effects of heterogeneity.

### Logistic regression analysis of isolated cell results

When heterogeneity was imposed in isolated SAN myocyte simulations, spontaneous APs stopped in a percentage of cells. To evaluate which parameters influenced this transition, we developed a logistical regression model that could be used to predict the cellular state (e.g. “spontaneous” or “dormant”) from a cell’s set of randomly-varied parameters, similar to previous studies on Ca^2+^ spark probability [26] or arrhythmic behavior [27]. In this statistical model, a logistic relationship is derived to relate the heterogeneous ionic conductances, placed in an input matrix, to the vector of cellular states, consisting of 1’s and 0’s for spontaneous and dormant cells, respectively. Each regression coefficient quantifies by how much, and in which direction, a model parameter needs to change to move a myocyte from the spontaneous to the dormant category.

### Mathematical modeling of electrical propagation throughout the SAN

We implemented a tissue model by connecting individual SAN cells through an intercellular resistance that represents the gap junctional channels. In this model, each cell is described by a system of ordinary differential equations that, integrated over time, yields the values of ionic concentrations and gating variables (state vector). In addition, the membrane potential is calculated through a partial differential equation since its value depends both on the individual cell and the neighboring cells in the tissue. Thus, the updating of the membrane potential is described by the following equation:

$$\frac{dV_{m}}{dt}=\frac{-(I_{ion}+I_{gj})}{C_{m}}$$

where *V_m_* is the membrane potential, *C_m_* is the cellular capacitance, *I_ion_* is the sum of all the ionic currents (dependent on the model), and *I_gj_* is the sum of the currents exchanged with the four neighboring cells. We define the sign of *I*_*gj*_ such that negative *I*_*gj*_ represents current flowing into a particular cell from its neighbors, which will depolarize that cell. To speed computation time, *V*_*m*_, which depends on *V*_*m*_ in neighboring cells, and the remaining state variables, which are specific to each cell, were updated separately. This allowed the updates to be computed in a massively parallel fashion using Graphical Processing Units, as described in more detail elsewhere [28]. Hardware and software specifications are provided in Table 1; model code is available at https://github.com/Eugenio95/2D_hetero_SAN_parallel_models.git.

**Table 1:** Hardware and software specification for model reproducibility.

Hardware			
**Workstation 1**		*Workstation 2*	
**Operating system**	Ubuntu 19.04	*Operating system*	Windows 10
**RAM**	64.0 GB	*RAM*	16.0 GB
**CPU**	16-core AMD Ryzen threadripper 2950x	*CPU*	Intel® Core™ i7-8700K
**GPU**	12 GB Nvidia Titan V	*GPU*	NVIDIA GeForce GTX 1060 6 GB
**Software**			
**Workstation 1**		*Workstation 2*	
**Simulation**	MATLAB R2019b; MATLAB GPU coder	*Simulation*	MATLAB R2020a; CUDA 8.0; Visual Studio 2015
**Integration**	Euler method (fixed step of 10 μs)	*Integration*	Euler method (fixed step of 10 μs)
**Analysis**	MATLAB R2019b Python 3.7	*Analysis*	MATLAB R2020a R-4.0.3

### Simulation protocols and conventions for model outputs

We considered a tissue formed of 2500 cells of equal size, arranged in a 50 x 50 matrix. Simulations were executed for a duration of 20 s. In addition to different amounts of cellular heterogeneity, we tested multiple levels of intercellular coupling from a resistance value of 10 MΩ (100 nS; strongly coupled cells) to 10,000 MΩ (0.1 nS; weakly coupled cells) [29,30]. The outputs of these simulations for each cell in the tissue were: membrane potential (V_m_), ionic current of each SAN cell (I_ion_), and gap junctional current (I_gj_). Additionally, I_net_ is defined as the sum of I_gj_ and I_ion_, reflecting the total net current of each cell. A negative I_net_ depolarizes the membrane, whereas a positive I_net_ hyperpolarizes it.

**Fig 2:**
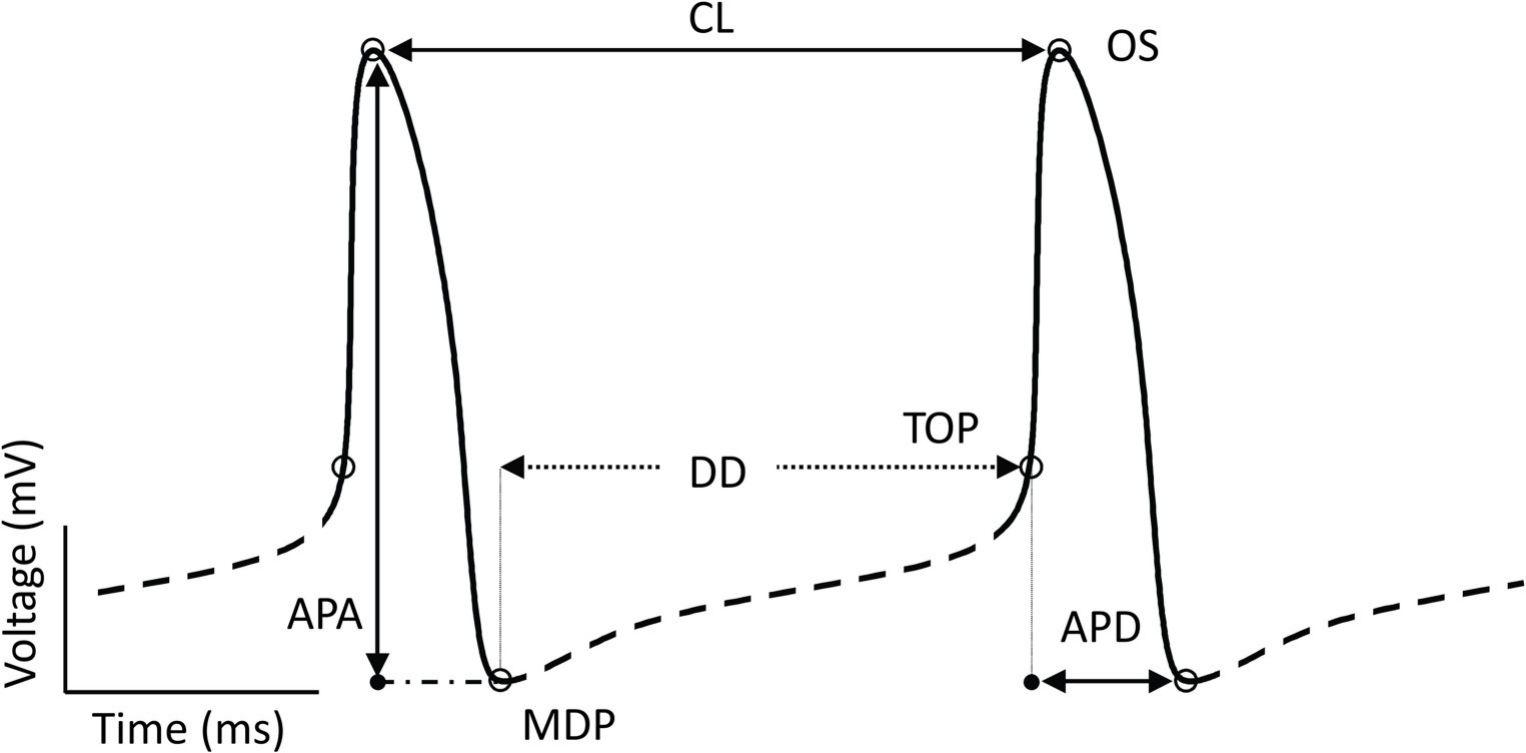
Features extracted from sinoatrial node AP simulations. A schematic AP trace is annotated with characteristic features. The full waveform is divided into the AP phase (solid line) and diastolic phase (dashed line) based on the criteria described. Critical voltages and durations are defined as labeled. Abbreviations: APA, action potential amplitude (mV); APD, action potential duration (ms); CL, cycle length (ms); DD, diastolic depolarization (ms); MDP, maximum diastolic potential (mV); OS, overshoot (mV); TOP, take off potential (mV).

From the AP trace (Fig 2) we defined maximum diastolic potential (MDP; in mV) as the minimum value of voltage during the cycle; overshoot (OS; mV) as the peak membrane voltage during the AP; and take-off potential (TOP; mV), as the voltage at the first time step during diastolic depolarization when $\frac{d^{2}V_{m}}{dt^{2}}$ exceeds 15% of the maximum $\frac{d^{2}V_{m}}{dt^{2}}$ [31]. These three outputs were then used to compute the metrics on which our analysis relied: DD (ms), or diastolic depolarization, is the phase of the AP between MDP and TOP; APD (ms), or action potential duration, is the time difference between TOP and the following MDP; CL (ms), or cycle length, is the time difference between two consecutive peaks; APA (mV), or action potential amplitude, is the difference in voltage between OS and MDP. Cells were classified as spontaneously beating when the following criteria were satisfied: (1) OS ≥ 0 mV; (2) MDP ≤ -40 mV; (3) at least 3 peaks in the final 5 s of simulation.

### Categorization of cells inside the tissue

**Fig 3:**
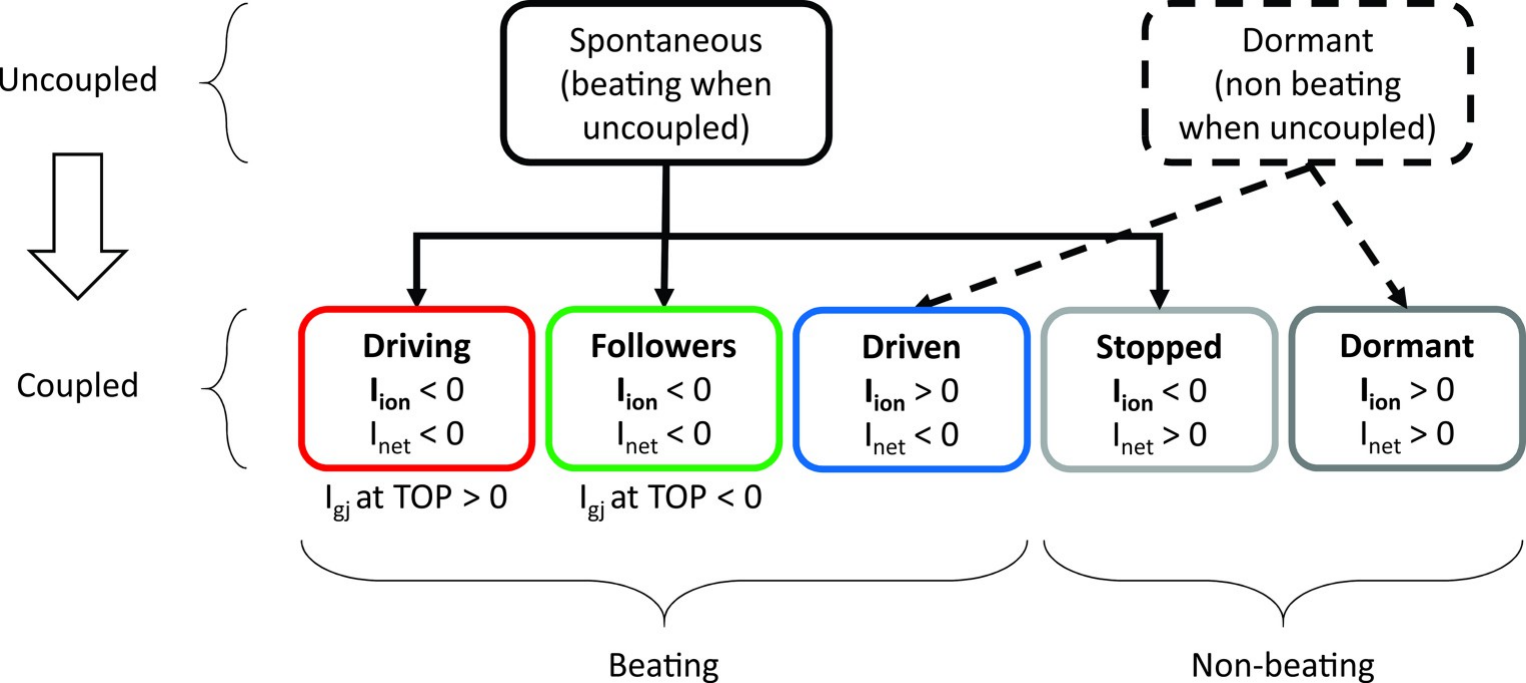
Cell categorization. Cells forming the tissue have been divided into different categories, depending on whether they exhibited action potentials, or not, under both coupled and uncoupled conditions. Alterations in intercellular coupling can cause an individual cell to switch categories, for instance from dormant at one value of coupling to driven at another value.

To better describe their behavior, the cells forming the 2D tissue were divided into categories. Initially, cells were defined as “spontaneous” or “dormant” depending on whether they showed rhythmic electrical activity when simulated in an uncoupled condition (R = ∞ MΩ). When a mixture of spontaneous and dormant cells is coupled in tissue, conditions may allow the dormant cells to exhibit action potentials. Understanding this concept requires the definition of subcategories, as illustrated in Fig 3, that capture different types of cellular behavior.

As schematized in Fig 3, based on their behavior when coupled within the tissue, “spontaneous” isolated cells could be further classified into: (1) “driving” if they continued to show rhythmic APs and had a positive (outward) I_gj_ at TOP, indicating that they reached threshold before adjacent cells and delivered current to their neighbors; (2) “followers” if, in spite of their spontaneous activity when uncoupled, they had a negative inward I_gj_ at TOP in the coupled condition, meaning that adjacent cells supplied current to assist their depolarization; (3) “stopped” if they did not show APs. On the other hand, “dormant” cells showed two different behaviors when coupled: (1) isolated dormant cells that started to beat thanks to coupling were called “driven,” whereas (2) cells that remained silent were termed “unexcitable.” Note that since “unexcitable” cells do not show APs under any condition, features such as TOP and DD are undefined for these cells. For “stopped” cells we calculated DD and TOP based on simulations performed in the uncoupled condition. This procedure allowed us to investigate the current generated and exchanged at corresponding time points when they were coupled.

## Results

### Increased heterogeneity causes failure of spontaneous beating in a fraction of isolated SAN cells

**Fig 4:**
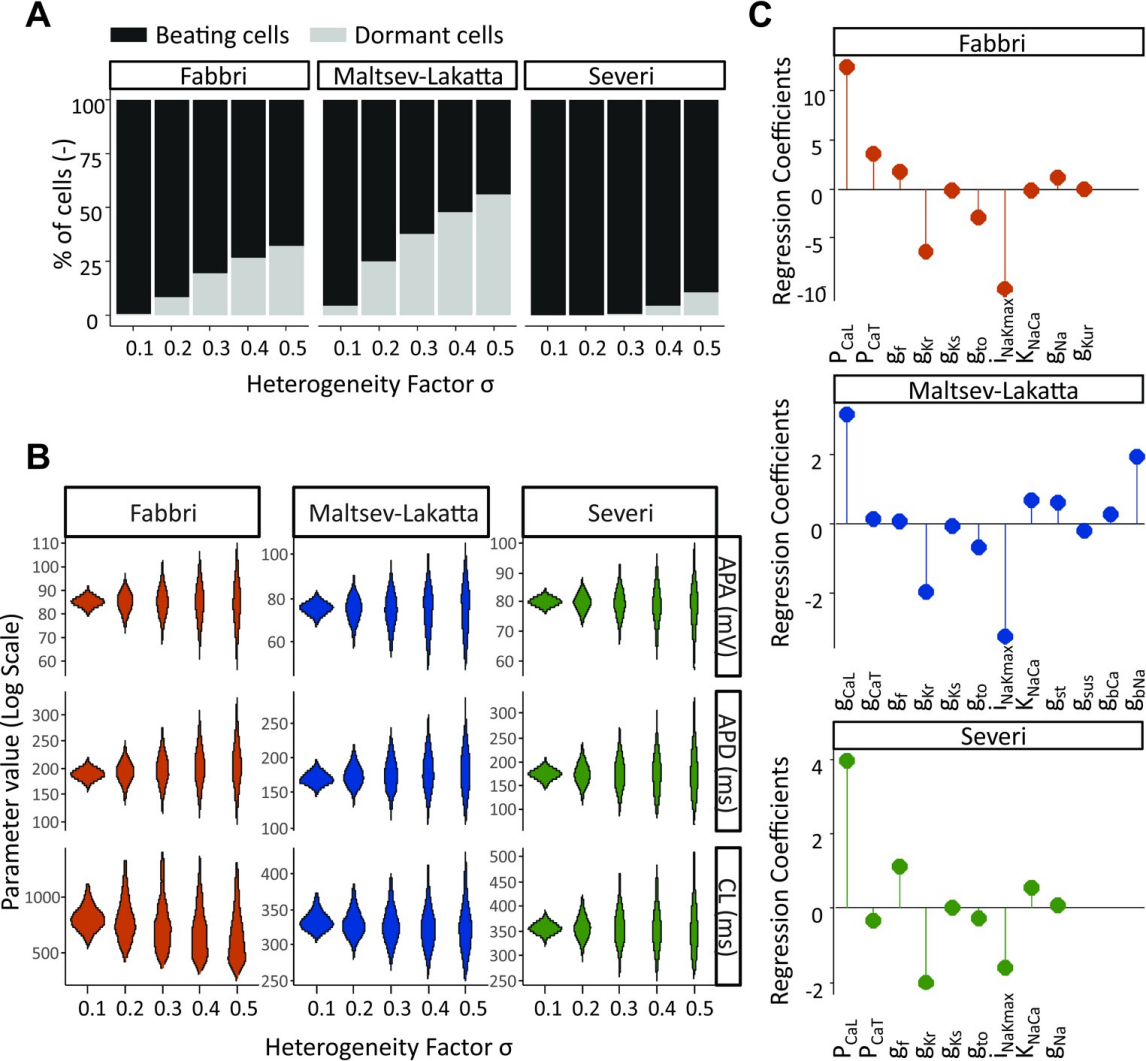
Modeling conductance heterogeneity using virtual populations of isolated SAN cells. (A) The effect of heterogeneous ionic channel expression on the automaticity of SAN cells was compared across models. In all three models the percentage of dormant cells rose with increasing levels of heterogeneity. (B) The effect of heterogeneity on the SA node AP properties was evaluated in spontaneously beating cells, by measuring cycle length (CL), AP amplitude (APA) and AP duration (APD) at varying σ levels. Outliers (values more than three median absolute deviations) were removed from distributions. (C) Logistic regression analysis was utilized to deduce which specific ionic currents across the three models are responsible for SA node cell’s automaticity. Positive values indicate that an increase in the parameter increases the probability of the cell to be spontaneously beating.

Following the approach described in the Methods, we introduced heterogeneity in the ionic currents underlying the APs of the 3 models studied [13–15]. Fig 4 illustrates the impact of heterogeneity on the excitability and electrical properties of the isolated SAN cells. It is evident from Fig 4A that at increasing levels of the heterogeneity factor σ, some cells within the population lose their automaticity. The percentage of dormant cells depends on the model, with the Severi model more resistant, and the Fabbri and Maltsev models more susceptible to increased variability in parameter values. In Fig 4B the AP metrics are summarized for the cells that retain their automaticity throughout various levels of heterogeneity. Across all models, there is a positive relationship between the level of heterogeneity and variability in AP amplitude, duration and frequency. Additionally, the Fabbri model shows a substantial decrease in the mean value of cycle length at increasing heterogeneity (-20% for σ = 0.5 vs σ = 0.1), while only much smaller decreases are seen in the other two models (-3% for Maltsev and -0.2% for Severi model). In Fig 4C, we examined which specific ionic currents were responsible for the automaticity. The results of the logistic regression analysis shown in this panel indicate how much each parameter needs to be altered to move the cell from the spontaneously beating to the silent group [26,27]. One notable difference between the 3 models is the background Na^+^ current I_bNa_, a current that is not even present in the Severi or Fabbri models, but which ranks as the third most important current in the Maltsev model. Despite this key difference, what is more notable is the consistency between the 3 models in terms of the relative importance of different currents in maintaining spontaneous activity. In all 3 cases, the L-type Ca^2+^ current, Na^+^-K^+^ pump, and rapid delayed rectifier K^+^ current I_Kr_ ranked as 3 of the most important parameters. Also notable is the relatively small regression coefficient corresponding to the “funny” current I_f_ in all 3 models. Considering that they were developed for different species (human vs. rabbit), from different data, and based on different hypotheses (Membrane clock vs. Ca^2+^-clock), this is not an obvious result.

### Well-coupled SAN tissues synchronize their behavior despite intercellular heterogeneity

**Fig 5:**
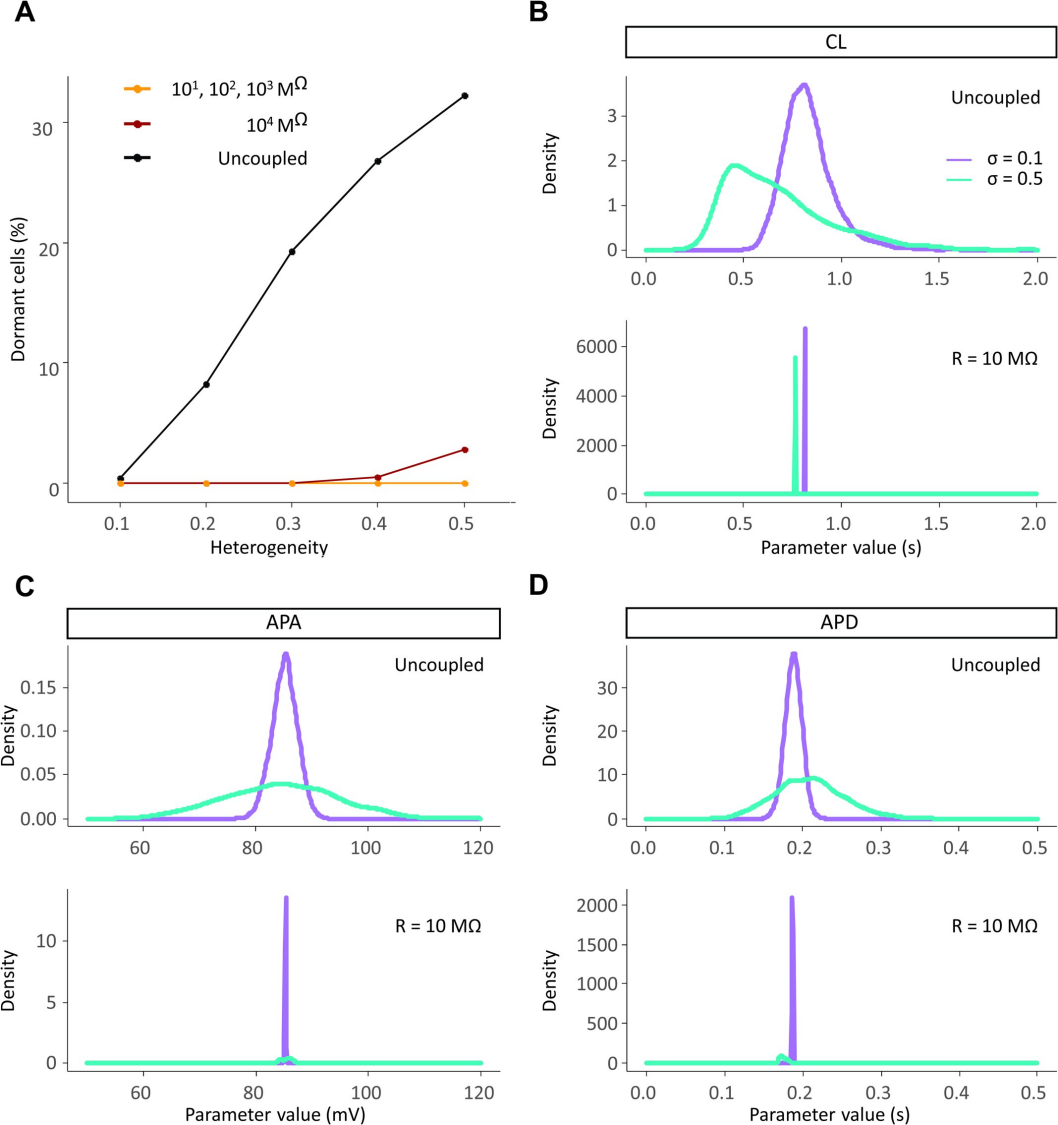
SAN cells synchronize their electrical properties when coupled in a tissue. (A) When coupled together heterogeneous SAN cells give rise to a spontaneously beating tissue. The only exception occurs at very high values of heterogeneity (σ equal to 0.4 and 0.5, with 0.5% and 2.8% of dormant cells respectively) and very high levels of intercellular resistance (R = 10,000 MΩ). (B-D) The population of cells synchronizes its AP metrics: cycle length (CL), action potential amplitude (APA) and action potential duration (APD) when well-connected in tissue.

Next we sought to investigate how variability between SAN myocytes influenced spontaneous beating at the tissue level. Since heterogeneity is known to be an important feature of the sinus node [8,32], we expected cells in well-coupled tissue to coordinate their beating and fire at a common rate. Fig 5 shows that this occurs in human tissue, which confirms previous findings obtained in rabbit multicellular simulations [5]. When cells are coupled in tissue, the percentage of dormant cells drops to near zero at all levels of heterogeneity (Fig 5A), and the CL shifts to a single value throughout the tissue (Fig 5B), which we define as tissue synchronization. Coupled SA nodal cells also mostly synchronize their action potential amplitudes (Fig 5C) and durations (Fig 5D), although some residual variability is observed when heterogeneity between cells is high (σ = 0.5). Thus intercellular coupling can act as a powerful synchronization mechanism in human SA node, as previously demonstrated in rabbit [5].

### Ionic current perturbations alter the relationship between gap junctional coupling and SAN automaticity

**Fig 6:**
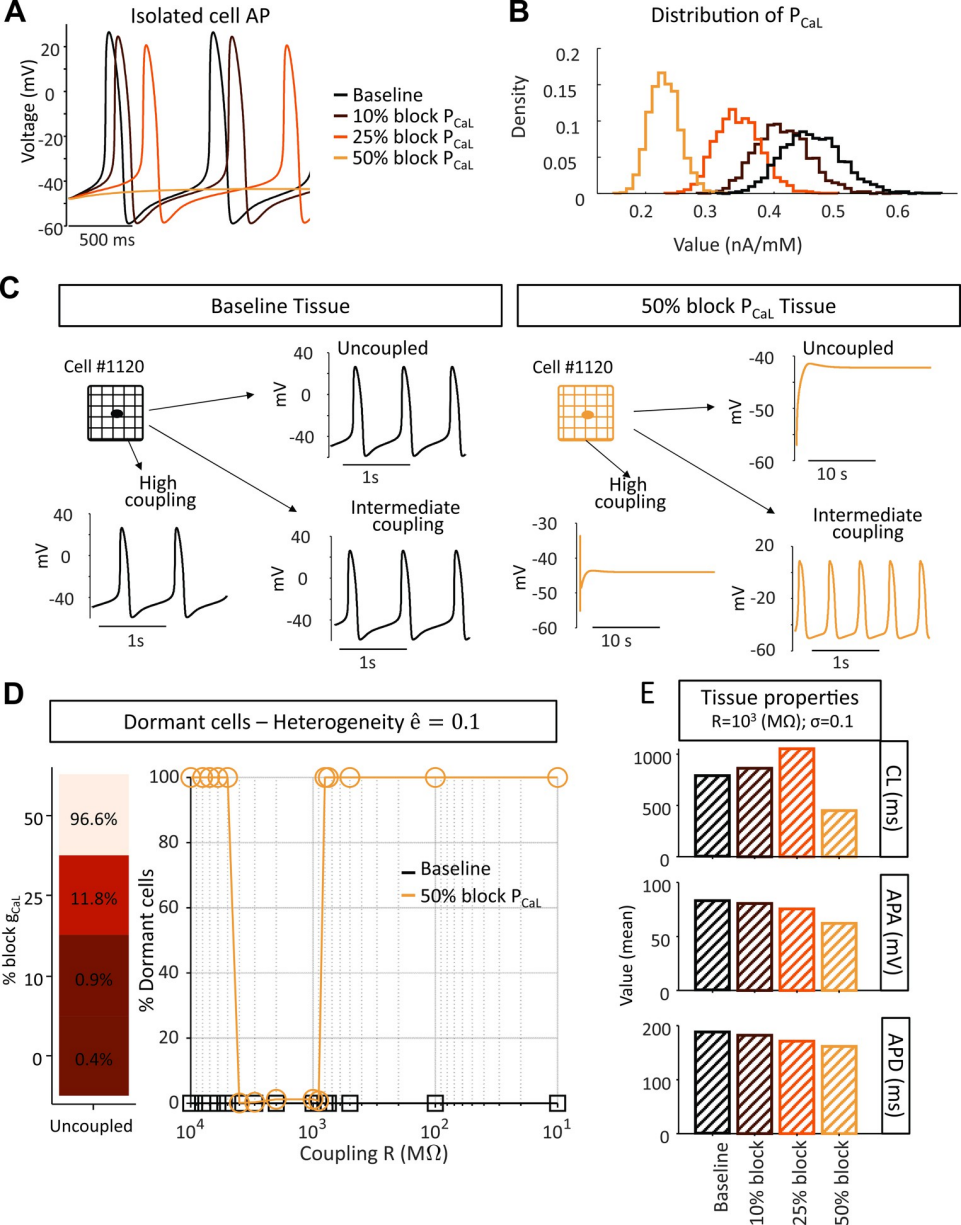
Certain coupling conditions restore automaticity to a prevalently dormant SA node tissue. (A) Effect of Ca^2+^ blockade in the Fabbri model with published parameters. (B) Distribution of P_CaL_ in the tissue at varying degrees of Ca^2+^ blockade (cellular heterogeneity factor σ equals 0.1). (C) Comparison of the electrical activity of a cell within the tissue (σ equals 0.1) before and after blockade of Ca^2+^ by 50%. (D) Dormant cells within the tissue beat at intermediate values of coupling. (E) Quantification of the average tissue CL, APA and APD at varying degrees of Ca^2+^ blockade.

The previous simulations suggested that strong intercellular coupling favors synchronization of SAN cells, since, for high levels of heterogeneity between isolated SAN myocytes, previously dormant cells exhibited synchronized beating in tissues. Next we explored the combined effects of heterogeneity and perturbations that inhibit spontaneous beating and are potential causes of SND. Fig 6A shows the impact of diminished I_CaL_ on the single cell AP of the Fabbri model. Blocking P_CaL_, the permeability controlling I_CaL_, by 10% or 25% causes a reduction in beating frequency and AP amplitude, and spontaneous beating stops at 50% block. Next, we analyzed the consequences of the same perturbations in heterogeneous tissue, which implies a shift in the distribution of P_CaL_ (Fig 6B). Unexpected results were seen, however, when these heterogeneous cells with reduced P_CaL_ were coupled in tissue. Fig 6C, for example, compares results at different levels of coupling in heterogeneous tissue (σ = 0.1), before (left) and after (right) 50% reduction of P_CaL_ in all cells. With normal P_CaL_, Cell #1120 exhibited spontaneous beating when uncoupled, and beat synchronously with the remainder of the tissue with both high and intermediate levels of intercellular coupling (left panels). The same cell, however, lost its ability to spontaneously beat when P_CaL_ was reduced by 50%. Surprisingly, however, this cell recovered its ability to beat at intermediate, but not at high, levels of intercellular coupling–i.e. certain levels of intermediate coupling encouraged SAN tissue automaticity. Generalizing to the whole tissue, Fig 6D shows that although the vast majority of cells (96.6%) did not beat spontaneously when electrically isolated, a middle range of coupling values (900 MΩ to 4000 MΩ), allowed these cells and the entire tissue to beat synchronously. The electrical properties of the tissue at different levels of P_CaL_ reduction and at intermediate coupling resistance (R = 1,000 MΩ) are quantified in Fig 6E. Blockade of I_CaL_ up to 25% caused the monolayer of SAN cells to beat at a lower frequency, but then at a higher rate when I_CaL_ is inhibited by 50% due to micro-reentry within the tissue (see S1 Movie).

**Fig 7:**
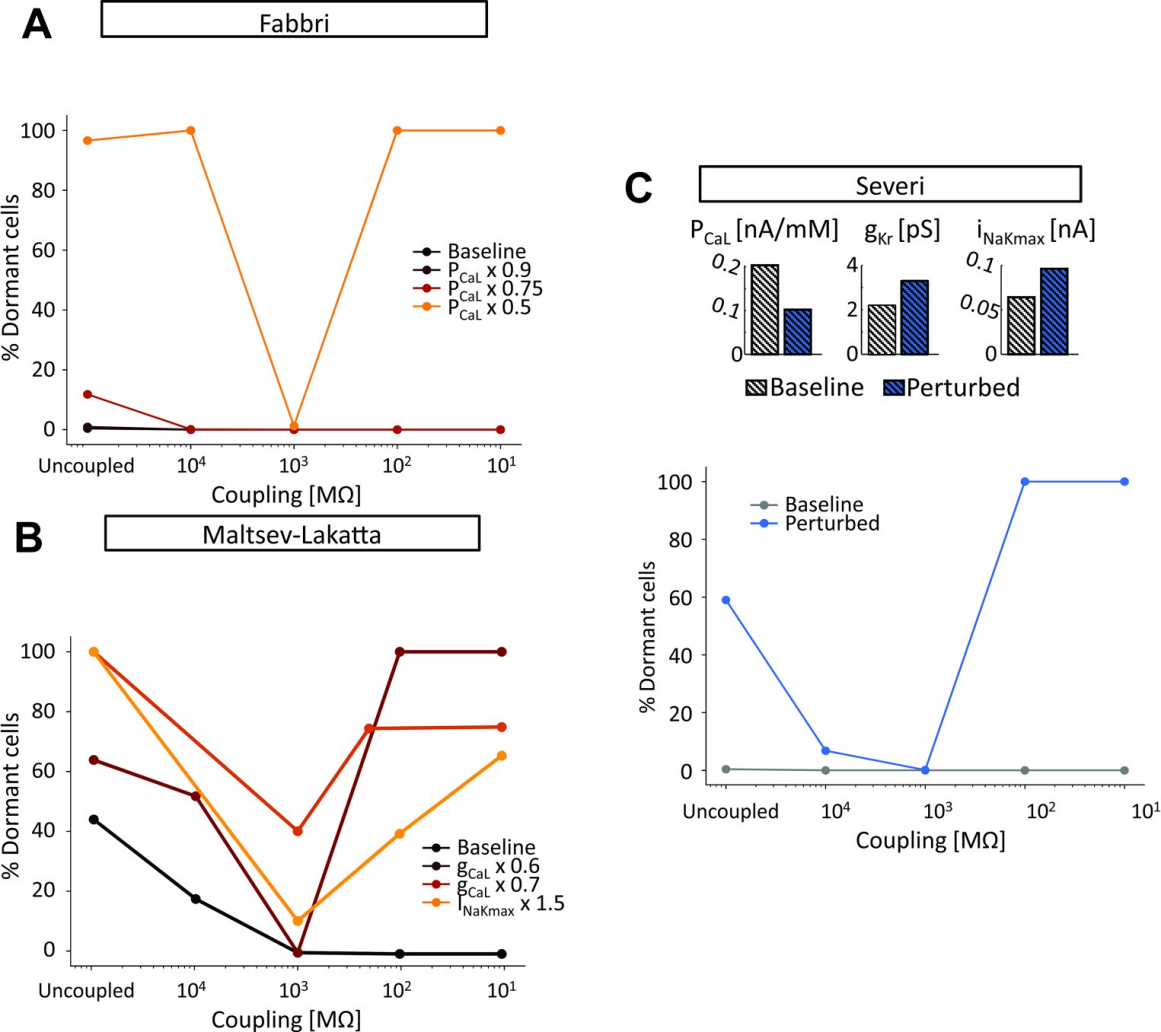
Pathophysiological changes in ionic currents lead to a pattern of tissue automaticity dependent on the degree of intercellular coupling. (A) Effect of L-type Ca^2+^ current (I_CaL_) perturbation in the Fabbri tissue model (σ equal to 0.1). (B) Effect of perturbation in I_CaL_ and Na^+^/K^+^ pump (I_NaK_) in the Maltsev-Lakatta tissue model (σ equal to 0.4). (C) Effect of combined I_CaL_, rapid delayed rectifier K^+^ current (I_Kr_), and I_NaK_ perturbation in the Severi tissue model (σ equal to 0.2).

Next we asked whether the protective effects of intermediate intercellular coupling were specific to the Fabbri model at 50% I_CaL_ reduction, or if this was a more general phenomenon. Fig 7 shows example results obtained in all 3 models where particular combinations of heterogeneity and ionic current perturbation led to synchronization of SA nodal tissue only at intermediate values of coupling. For example, when combined with heterogeneity, a 50% reduction of I_CaL_ in the Fabbri model (Fig 7A), either an increase in I_NaK_ or a decrease in I_CaL_ in the Maltsev model (Fig 7B), or a combination of 3 parameter changes in the Severi model (Fig 7C), all led to failure of spontaneous beating with strong intercellular coupling, successful propagation through the tissue at intermediate levels of coupling, and a substantial percentage of non-beating myocytes (> 60%) when cells were completely uncoupled. These results therefore suggest that intermediate coupling may enable the SA nodal tissue to beat spontaneously under a range of conditions that will lead to failure when coupling between myocytes is strong. S2 and S3 Movies show the patterns of electrical activity in representative simulations from the Maltsev-Lakatta and Severi tissue models, respectively.

### Clusters of beating cells can drive AP propagation over a range of coupling strengths

**Fig 8:**
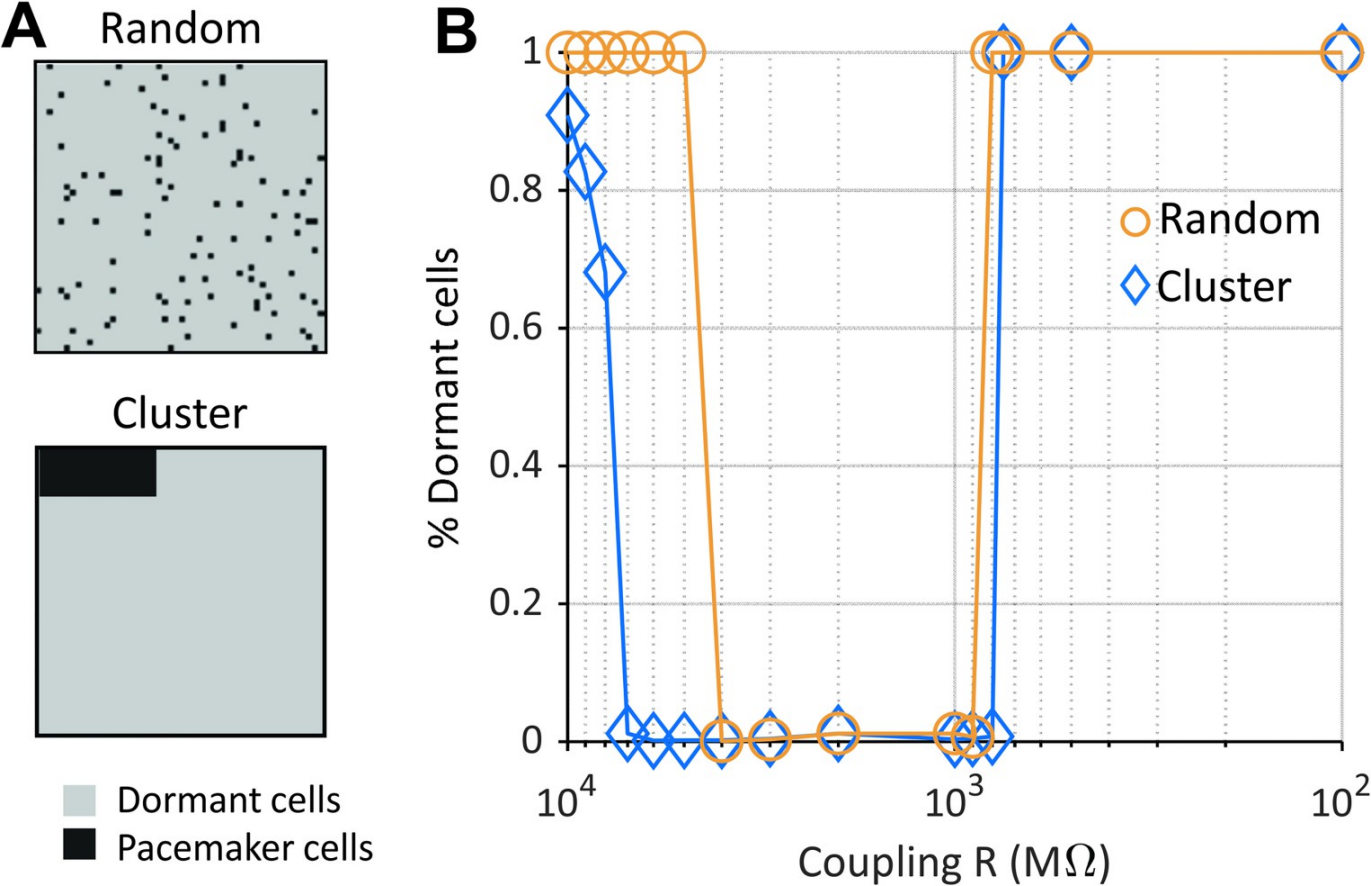
A small cluster of pacemaker cells can drive a prevalently dormant tissue. (A-top) (A) In the random tissue configuration dormant and pacemaker cells are interspersed in the matrix. (A-bottom) In the cluster configuration pacemaker cells are confined to a small portion of the matrix surrounded by dormant cells. Here dormant cells are cells that fail to depolarize after inhibition of I_CaL_ by 50%. (B) The range of intercellular coupling compatible with AP generation and entrainment, i.e. reduced percentage of dormant cells, is wider in the cluster tissue configuration compared to the random. Results shown here were obtained with Fabbri human model (σ equal to 0.1).

The results shown in Fig 6 demonstrated that a small number of spontaneously beating SAN myocytes could, at certain coupling strengths, drive propagation in the entire tissue. In that case, however, cells were distributed randomly throughout the tissue, whereas anatomical studies suggest clustering of similar cells in different regions of the SA node [33,34]. We therefore tested the effects of placing all spontaneously-beating SA nodal cells within a defined cluster (Fig 8A). Results show that the clustered myocytes can drive propagation through the rest of the tissue over a wider range of coupling strengths, compared with the randomly-distributed, spontaneously-beating cells (Fig 8B). These results therefore suggest that pacemaker cells, when co-localized in a subregion of the node, may be protected from the influences of neighboring cells of a different type [35].

### Intermediate coupling encourages tissue beating due to interactions between driving cells and dormant cells

Results presented thus far suggest that to understand the mechanisms of excitability in the overall tissue, we need to take a closer look at what occurs in the vicinity of the few pacemaker cells present in the tissue. In particular, we are interested in uncovering how, under conditions when a majority of cells do not exhibit spontaneous beating, a small percentage of cells is able to drive tissue depolarization within a narrow range of intercellular coupling.

**Fig 9:**
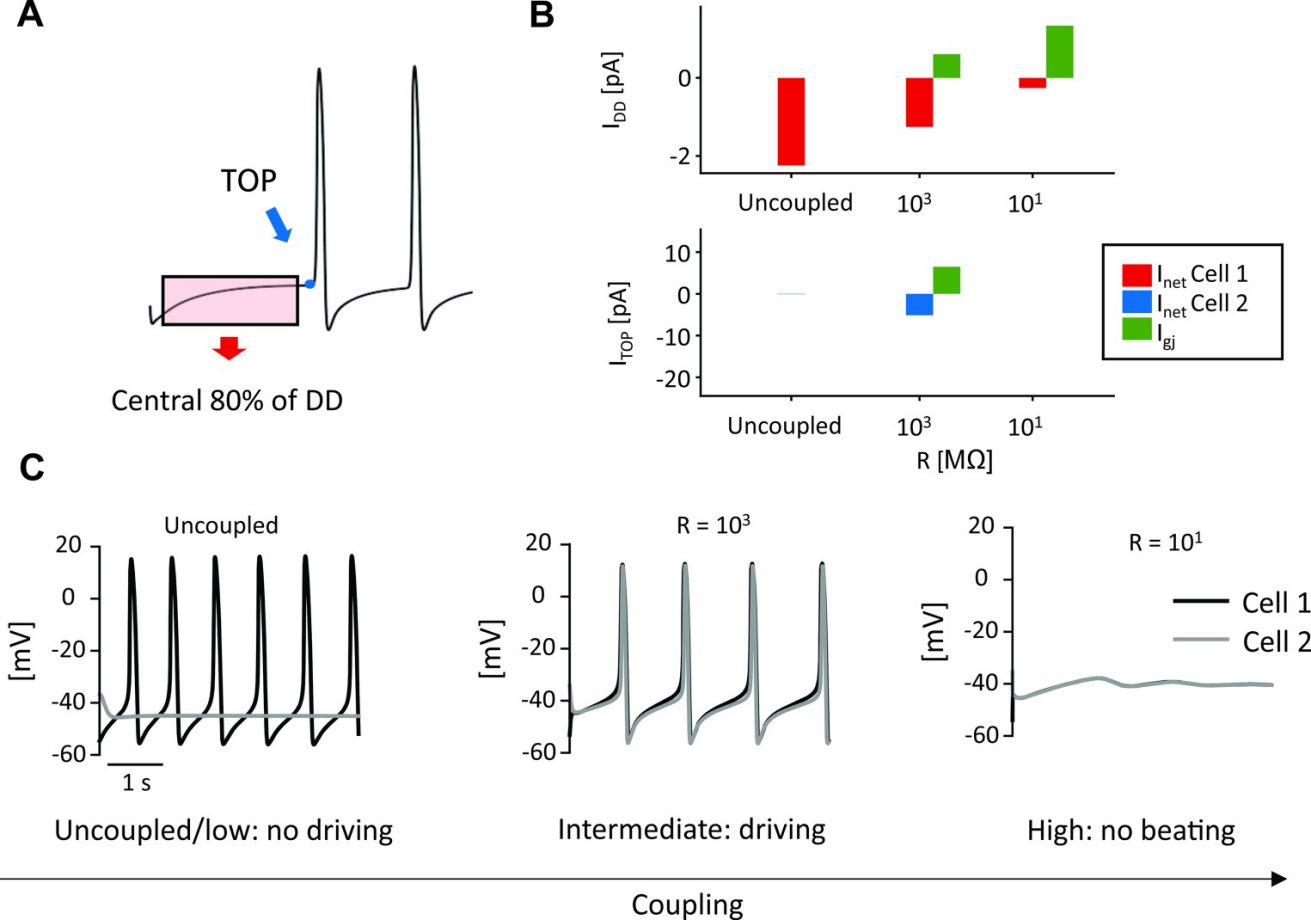
Coupling between a spontaneous cell and a dormant cell. (A) Average I_net_ and I_gj_ were extracted from the central 80% portion of the first occurrence of DD (from the beginning of the simulation to the first TOP); TOP I_net_ and I_gj_ were sampled at the time of the TOP. (B) I_net_ and I_gj_ trends during diastole (top) for Cell 1 (spontaneous) and at TOP (bottom) for Cell 2 (dormant) with respect to different degrees of cellular coupling. I_gj_ is plotted in green for Cell 1 in both panels (the positive sign indicates an outward current, supplied to Cell 2). (C) Behavior of the two cells depending on coupling: both cells are beating periodically only for intermediate coupling values.

To investigate this question, we performed simulations with a spontaneously beating and a dormant cell (both extracted from tissue with σ = 0.3, 50% P_CaL_ reduction). From the simulation results of the two cells, we computed the average I_net_ during the central portion of the DD and, when action potentials occurred, I_net_ at the TOP (Fig 9A). Plots of these quantities over a range of coupling resistances (Fig 9B) help to explain why the spontaneously beating cell (Cell 1) is only able to drive the dormant cell (Cell 2) at intermediate coupling values. When the coupling between the two cells is strong (R = 10^1^ MΩ), the dormant cell can suppress action potentials in the cell that would otherwise beat spontaneously (Fig 9C, right). This occurs because the large gap junctional current through the low resistance junction results in a small magnitude of diastolic I_net_ in the spontaneous cell. Under these conditions, TOP I_net_ is undefined since neither cell reaches TOP. With reduced coupling between the two cells (R = 10^3^ MΩ) gap junctional current between the two cells is reduced, which allows a larger magnitude of diastolic I_net_ in Cell 1 (Fig 9B, top). This enables Cell 1 to reach its TOP and fully activate its inward current, thereby supplying enough current to Cell 2 for it to reach its TOP (Fig 9B, bottom) and fire an AP (Fig 9C, middle). Finally, when the coupling between the cells is reduced further (R = 10^4^ and higher), a large inward diastolic I_net_ in Cell 1 is able to bring this cell to TOP, but the small magnitude of coupling current means that Cell 1 is unable to drive beating in Cell 2. Thus, intermediate values of coupling represent a “sweet spot” at which the spontaneously beating cell and the dormant cell can be synchronized.

To further support this view, the same analysis of I_net_ was applied to the whole 2D tissue (σ = 0.3, 50% P_CaL_ reduction). To understand this significantly more complex situation, cells were divided into categories based on their behavior, as explained in the Methods section. In these simulations, the initial condition for each spontaneous cell was set as state vector at the MDP in the uncoupled condition, and initial conditions for dormant cells were set at those of the spontaneous cell with the most depolarized MDP.

As with the cell pair, strong coupling (R = 10^1^−10^2^ MΩ) allows dormant cells to suppress electrical activity in spontaneous cells (

**Fig 10:**
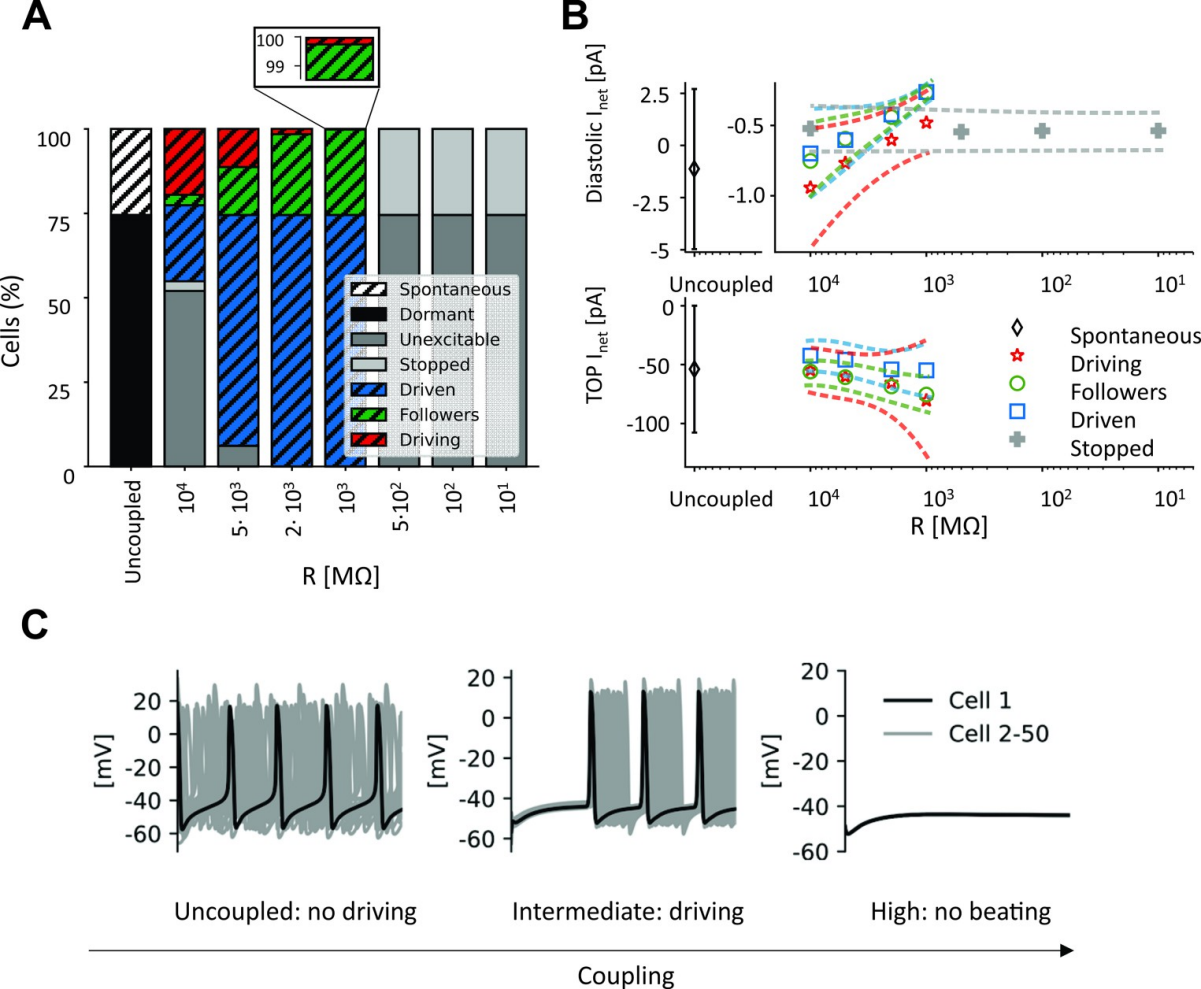
Coupling spontaneous cells with dormant cells inside a tissue. (A) Percentages of cells composing each category at different degrees of intercellular coupling. (B) I_net_ trends during diastole (top) and at TOP (bottom) with respect to different degrees of intercellular coupling for every cell category. Average value (symbol) ± standard deviation (dashed line). (C) Electrical activity of 50 cells (4^th^ column of the 2D tissue matrix) when they are coupled with different intercellular resistances.

Fig 10C, right) by draining current during the diastolic phase. Thus, TOP is not reached and the entirety of the tissue becomes “stopped” or “dormant” (right side of Fig 10A). With reduced coupling (R = 10^3^ MΩ), spontaneous cells retain a larger fraction of diastolic I_net_ which allows them to reach the TOP. The first cells to reach TOP are classified as “driving,” since they supply current to the other cells in the tissue, which are either “followers,” if they beat spontaneously, or “driven,” if they are otherwise dormant. A further reduction in the coupling (R = 10^4^ and higher, left part of Fig 10A) allows more cells to reach the TOP on their own, but these spontaneously-beating cells are able to only drive a small percentage of the remainder of the tissue, due to reduced gap junctional currents between myocytes. Thus, under conditions of reduced excitability, the magnitudes of currents flowing between spontaneous and dormant cells determine whether the tissue can become entrained.

## Discussion

In the present study, we investigated how different levels of cellular heterogeneity and intercellular coupling influenced human and rabbit SAN pacemaking. We simulated both healthy tissue and conditions of reduced excitability that were meant to approximate SND arising from diverse causes. Results showed that although increased cellular heterogeneity leads to a growing fraction of cells losing automaticity, intercellular coupling allows for synchronous and rhythmic activity in the whole tissue. Of note, this remained true for nearly all combinations of heterogeneity and coupling, highlighting the robustness of beating in nodal tissue. When we simulated diseased conditions by increasing or decreasing levels of fundamental ionic currents, the SAN tissue could fail to depolarize spontaneously. However, even under these extreme conditions, intermediate values of gap junctional resistance could rescue SAN electrical activity, and simulations provided mechanistic insight into this unusual phenomenon. This behavior was seen in all 3 models that we examined [13–15], and with different causes of reduced cellular excitability, suggesting that it may be a general property of SAN entrainment rather than specific to particular circumstances.

### Comparison with previous computational SAN studies

Mathematical modeling has been employed as a tool to understand the mechanisms of SAN coupling and entrainment for more than two decades. Early studies [7,36] demonstrated how simulations of SAN pacemaker activity in models of coupled cells can provide insights and encourage new hypotheses about cardiac electrical conduction. Combined with animal experiments, modeling has been instrumental in developing our understanding that the heartbeat is likely to be dictated by the mutual entrainment of multiple spontaneously beating cells that synchronize their activity. Over the years, many investigators developed models to further describe the role of mutual entrainment of heterogeneous cells in the generation of the pacemaker activity. For instance, Oren and Clancy [37] showed that connections between the SAN and the atrium might be sufficient to impart the different features of peripheral SAN compared with central SAN APs. Conversely, Inada and colleagues [30] argued for the necessity of gradual changes in cell size, ionic current densities, and intercellular coupling from center to periphery. In particular, they suggested that the expression of Na_v_1.5 and Cx43 in the periphery of the SAN might be fundamental for driving propagation to the atrium. Additional relevant insights were obtained by Gratz et al. [5], who studied interactions between ion channel conductances and intercellular coupling and found that the factors determining synchrony depended on whether this was defined by a metric based on activation times or one based on peak voltages. This study [5] is especially relevant to our work, as these authors examined synchronization of heterogeneous SAN tissue over a range of coupling strengths. Also pertinent is a recent study by Maltsev et al. [38], who examined tissue under conditions where the average cell was close to the border between spontaneously beating and dormant, finding that heterogeneity between myocytes enhanced the firing stability of the tissue. Our work builds on this prior research by perturbing myocytes in a heterogeneous population and demonstrating that a small percentage of spontaneously-excitable cells can sometimes be sufficient to drive the remainder of the tissue.

### Modeling insights into the physiology and pathophysiology of the SAN

We simulated the effects on SAN automaticity of both physiological heterogeneity in ionic current densities and pathological changes to these currents. The results showed that this heterogeneity is compatible with synchronization of a large monolayer of either human or rabbit SAN cells. Moreover, we suggest that under conditions of reduced coupling between nodal cells, this heterogeneity helps to impart remarkable resilience that allows for AP entrainment even in the presence of pathological changes in the cellular electrical properties.

Sinus Node Disease (SND), also referred to as Sick Sinus Syndrome, is a general term that encompasses SA nodal dysfunction resulting from a wide variety of causes. Most cases of SND are acquired and associated with aging [39], but several congenital forms caused by mutations in ion channels or associated proteins have also been described [34,40]. Normal aging, which frequently produces a reduction in heart rate, is also associated with decreases in expression of peripheral Na^+^ channels [41,42] and Cx43 [43]. Heart failure (HF), chronic atrial fibrillation and cell apoptosis [44] can also contribute to structural and electrical remodeling of the node and SAN dysfunction. Given the complexity involved in different types of SND, our goal was to broadly study conditions that caused some cells to lose automaticity, rather than any particular pathological state. Accordingly, we inhibited automaticity by altering different ionic currents in the 3 models (see Fig 7).

Whatever the cause of dysfunction, our results show that heterogeneity and intercellular coupling are important factors in allowing SAN tissue to continue to exhibit spontaneous beating in the face of potentially pathological perturbations. Our simulations revealed that specific coupling strengths, falling in the range 900–4000 MΩ, or 0.25–1.1 nS when expressed as conductances (Fig 6D), allow the tissue to beat even under conditions where many cells no longer spontaneously fire. Comparing this range of coupling strengths to the existing literature, we find that it sits at the low end of previously reported values. For instance, experimental studies on rabbit SAN suggested that 0.5 nS would allow for frequency entrainment and 10 nS for waveform entrainment [3]. Other computational investigations have employed intercellular resistances of 7.5 nS [37] and 25 nS [30], whereas experiments have estimated values such as 0.6–25 nS [29] and 2.6±0.6 nS [45]. Values of intercellular coupling may be non-uniform across the SAN if different connexin isoforms are expressed in different SAN regions. Thus, the protective range of coupling that our simulations identified, which became relevant under simulated pathological conditions, is consistent with the fibrosis observed under pathological conditions [21,22], which is likely to be associated with reduced coupling between SAN cells. One could even speculate that fibrosis, remodeling of gap junctions, and decreased connexin expression in SND may help to protect the SAN from failure.

Naively, one might expect that stronger coupling between SA nodal myocytes will be beneficial, since this will lead to faster propagation and enhanced synchronization of the cells within the node. Although our results are consistent with this idea under normal conditions, our findings also highlight a potential advantage of reduced coupling–namely that this can impart the tissue with greater resilience under conditions that impair spontaneous beating in individual myocytes. Indeed, it is remarkable that under particular conditions, fewer than 10% of the cells in the tissue can drive electrical activity in the remaining 90% of myocytes that do not.

### The protection provided by intermediate coupling: AP vs. DD intercellular interactions

To attempt to explain the protective range of coupling strengths under pathological conditions, (Figs 6 and 7), we formulated a hypothesis based on the concepts of tonic and phasic entrainment that are well-established in the SAN literature [3,46]. What differentiates our results from these previous ideas is that in our simulations these two types of interaction not only regulate SAN synchronization, but also determine the presence of spontaneous beating inside the tissue. In other words, spontaneous cells manage to drive dormant cells only if two conditions are satisfied. First, spontaneous cells have to reach the take-off potential. Second, they have to supply enough current to the neighboring dormant cells. In this scenario, coupling resistance becomes the most critical parameter, since deviations in either direction can cause spontaneous firing of the tissue to fail. If resistance is too low, dormant cells will hyperpolarize the spontaneous ones during the DD phase, preventing them from reaching the threshold for AP firing. On the other hand, if coupling resistance is too high, spontaneous cells will not supply enough current to depolarize dormant cells. However, intermediate values of coupling guarantee that both conditions are satisfied. During diastole, when the voltage difference is low, I_gj_ is negligible, whereas during the upstroke, I_gj_ increases and allows dormant cells to depolarize (Figs 9 and 10). Although the cell types are different, this general phenomenon resembles the propagation of ectopic beats in ventricular tissue, where reduced coupling encourages propagation by inhibiting dissipation of depolarizing current [47,48].

### Are dormant cells present inside the sinoatrial node?

Given the numerous mechanisms that interact to produce SA nodal pacemaking at the cellular level [49,50], it was not especially surprising that heterogeneity in ionic current properties caused a percentage of cells to cease beating spontaneously. Although it seems reasonable to ask whether this behavior is realistic or an artifact of the modeling, recent studies strongly suggest that dormant cells do indeed exist, both in isolated cell studies and within intact SA nodal tissue. A combined experimental and computational work published in 2018 [8] reported that about half of SAN cells isolated from guinea pig hearts did not exhibit spontaneous APs, although many of these cells recovered spontaneous beating when β-adrenergic signaling was stimulated with isoproterenol. A limitation of that study, however, is that results could have been influenced by the enzymatic dissociation procedure used to isolate individual cells. More recent studies, from that group and others [9,51,52], have confirmed the existence of dormant SA nodal cells in tissue under a variety of conditions. Our results, along with similar recent modeling studies [38], demonstrate that when dormant cells are coupled with a minority of spontaneous cells, the tissue can exhibit stable electrical activity even in the absence of sympathetic stimulation. Our results also suggest that relatively large percentages of dormant cells can indeed be consistent with normal pacemaker function at the tissue level due to the protective effects of heterogeneity and intercellular coupling. An excessive presence of dormant cells nevertheless poses a threat to SAN function, since these conditions restrict the coupling range in which rhythmic electrical activity can be generated. This highlights the perils of pathologies such as SND that depress SAN cellular excitability.

### Limitations and future developments

Although the modeling strategy we used in this study allowed us to investigate tissue automaticity under a wide range of conditions, several limitations of our approach should be mentioned. First, the cellular heterogeneity was represented as random differences in ion channel expression between cells, and we did not consider gradients across the tissue in cell type, size, or shape. Several different types of myocytes have been proposed to exist within the SA node [22,34], and non-myocyte cell types such as fibroblasts, atrial cells and adipocytes have been hypothesized to play important roles [21,33,53], and we did not examine these possibilities. Another structural simplification is the idealized geometry represented by a square sheet, far from the 3D banana-shaped anatomy of the SAN [22,33]. Our tissue, which comprised 2500 cells, is comparable in size to the rabbit SAN (about 5000 cells [54]), but represents only a fraction of the human SAN. An additional limitation is that the isolated cell models we used are appropriate for for tissue simulations of electrical propagation, but not well-suited for local calcium release events that contribute to normal pacemaking and can appear even in dormant cells [8,55]. More complex cellular models that consider stochastic gating of intracellular release channels [56] are required to simulate these local phenomena. These limitations can be addressed in future work to shed additional light on mechanisms of SAN pacemaking.

## Conclusions

In conclusion, we have shown how multiscale mathematical modeling can be used to gain insight into the importance of cellular heterogeneity and intercellular coupling for efficacious cardiac entrainment. Previous multicellular studies have shown that synchronization of heterogenous cells is responsible for the SAN pacemaker function in rabbits [5,38]. Our data confirmed that the same phenomenon occurs in a two-dimensional model of the human sinoatrial node. In addition, our study suggests that certain degrees of intercellular coupling make the sinoatrial node resistant to ionic perturbations that might be provoked by mutations and/or drug therapies.

## Supporting information

S1 Movie Dynamic SAN activity in the Fabbri model. Movie illustrates electrical activity in the SAN tissue using the Fabbri model under conditions close to propagation failure. Simulation performed with P_CaL_ reduced by 50%, R = 1000 MΩ, σ = 0.2.
(AVI)

S2 Movie Dynamic SAN activity in the Maltsev model. Movie illustrates electrical activity in the SAN tissue using the Maltsev model with I_NaK_ increased by 50%, R = 1000 MΩ, σ = 0.4.
(AVI)

S3 Movie Dynamic SAN activity in the Severi model. Movie illustrates electrical activity in the SAN tissue using the Severi model with ionic current perturbations as shown in Fig 7, R = 1000 MΩ, σ = 0.2.
(AVI)
